# The complete plastome of *Glandora prostrata* subsp. *lusitanica* (Samp.) D.C.Thomas (Boraginaceae), the first chloroplast genome belonging to the *Glandora* genus

**DOI:** 10.1080/23802359.2023.2175976

**Published:** 2023-02-15

**Authors:** Inês Carvalho Leonardo, Adriana Alberti, France Denoeud, Maria Teresa Barreto Crespo, Jorge Capelo, Frédéric Bustos Gaspar

**Affiliations:** a iBET, Instituto de Biologia Experimental e Tecnológica, Oeiras, Portugal; b ITQB-NOVA, Instituto de Tecnologia Química e Biológica António Xavier, Universidade Nova de Lisboa, Oeiras, Portugal; c Génomique Métabolique, Genoscope, Institut François Jacob, CEA, CNRS, Univ Évry, Université Paris-Saclay, Évry, France; d ECOCHANGE, CIBIO-InBIO – Research Centre in Biodiversity and Genetic Resources, Universidade do Porto, Vairão, Portugal; e INIAV, Instituto Nacional de Investigação Agrária e Veterinária I.P., Quinta do Marquês, Oeiras, Portugal

**Keywords:** *Glandora prostrata* subsp. *lusitanica*, Boraginaceae, complete chloroplast genome, Illumina MiSeq sequencing, phylogenetic analysis

## Abstract

*Glandora prostrata* (Loisel.) D.C.Thomas (Thomas et al., 2008), besides being a common plant of western and south-western Europe and north-western Africa, is a species with a wealth of reported uses in traditional and folk medicine. The chloroplast genome of *Glandora prostrata* subsp. *lusitanica* (Samp.) D.C.Thomas (Thomas et al., 2008) isolate BPTPS049 described in this study is the first publicly available complete plastome belonging to the *Glandora* genus. The chloroplast genome (GenBank accession number: ON641304) is 150,041 bp in length with 37.5% GC content, displaying a quadripartite structure that contains a pair of inverted repeat regions (25,833 bp each), separated by a large (81,222 bp) and small (17,153 bp) single-copy regions. It has 131 annotated genes including 86 protein-coding genes, 37 tRNA genes, and eight rRNA genes. The phylogenetic analysis performed confirms that *G. prostrata* subsp. *lusitanica* is placed under the Boraginaceae family, which belongs to the Boraginales order. This study will contribute to conservation, phylogenetic, and evolutionary studies that comprise this traditional species relevant to the landscape of aromatic, medicinal, and condiment plants from Portugal.

## Introduction

*Glandora* D.C.Thomas, Weigend & Hilger (Thomas et al., 2008) is a genus of flowering plants (angiosperms) in the Boraginaceae family, that includes eight species, distributed primarily in western and south-western Europe (Portugal, Spain, France, Italy, and reaching Greece) and in north-western Africa (Morocco and Algeria). This genus was recently segregated from the more widespread *Lithodora* L. based on molecular methods (Thomas et al. 2008) and micromorphological features (Ferrero et al. 2012). Plants of both genera are known as ‘gromwells’ and are found in shrubs or rocky environments.

*Glandora prostrata* (Loisel.) D.C.Thomas (Thomas et al., 2008) is a common plant of heathland and scrubland of which a wealth of uses in traditional and folk medicine have been reported (anti-bacterial, anti-viral, anti-inflammatory, anti-rheumatic, anti-spasmodic, anti-pyretic, anti-tussive, anti-dermatosic, analgesic, and sedative) (Novais et al. 2004). There are few pharmacological studies using *G. prostrata* nevertheless the toxicity of its phenolic compounds to human-colorectal and gastric adenocarcinoma cell lines has been referred by Fernandes et al. (2017). Among the different *G. prostrata* ecotypes found in the wild, *Glandora prostrata* subsp. *lusitanica* (Samp.) D.C.Thomas (Thomas et al., 2008) was selected to be further explored.

## Materials and methods

**Figure 1.**
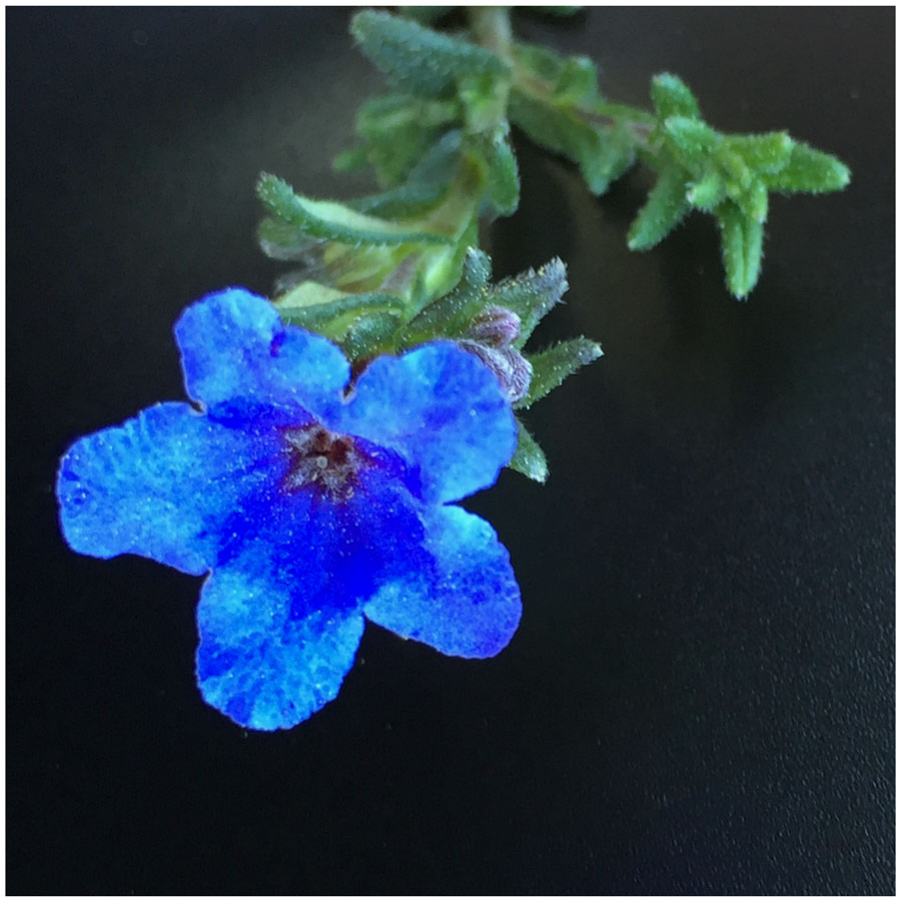
Picture of *Glandora prostrata* subsp. *lusitanica* isolate BPTPS049 on the day of its collection from a wild population in the dunes of the Beja municipality (Vila Nova de Mil Fontes) in Portugal (collection date: 19 March 2019; location: 37.71500 N, 8.78361 W). BioSample: SAMN28118496.

The plant material of *G. prostrata* subsp. *lusitanica* (BioSample: SAMN28118496; Figure 1) analyzed in this study was collected from a wild population in the dunes of the Beja municipality (Vila Nova de Mil Fontes) in Portugal (collection date: 19 March 2019; location: 37.71500 N 8.78361 W). This plant material was identified as isolate BPTPS049 with a specimen being conserved at the LISE Herbarium (INIAV, Oeiras, Portugal; Jorge Capelo: jorge.capelo@iniav.pt; Figure 1S) under the voucher LISE: 96377 (identified by: Jorge Capelo).

Young leaves were selected from the collected specimen, frozen in liquid nitrogen immediately after collection, and stored at −80 °C until further processing. Total genomic DNA was extracted using an adaptation of the Doyle and Doyle (1987) methodology. The obtained DNA was sent to Genoscope (Évry, France) for sequencing. DNA was first sonicated using the Covaris E210 Focused Ultrasonicator instrument (Woburn, MA), and then libraries were prepared with the NEBNext Ultra II DNA Library Prep Kit (New England Biolabs, Ipswich, MA). Finally, sequencing was performed using 151 base-length read chemistry in a paired-end flow cell on the Illumina NovaSeq 6000 sequencing platform (San Diego, CA).

The about 24 million high-quality paired-end reads obtained (SRA: ERR10047929) were used to assemble the complete chloroplast genome (sequence coverage: 993×) using the GetOrganelle pipeline (v1.7.3.1) (Jin et al. 2020). The pipeline was used following the typical recipe suggested for Embryophyta plant plastome assembly (https://github.com/Kinggerm/GetOrganelle) while setting the flags ‘-max-reads’ and ‘-reduce-reads-for-coverage’ to 25 million and one thousand, respectively (see supplemental material for additional details). The plastome annotation was performed using the GeSeq tool (Tillich et al. 2017) using the default parameters and the provided 3rd party stand-alone annotator Chloë (v0.1.0). A subsequent manual curation of the obtained annotations was performed using Geneious Prime 2022.0.1 (https://www.geneious.com) while comparing with the results obtained from a BLAT (BLAST-like alignment tool) (Kent 2002) search also using the GeSeq tool (with protein, rRNA, tRNA, DNA search identities set to 90%; see supplemental material for additional details).

The dataset used for the phylogenetic analysis was obtained from GenBank (accession date: 1 June 2022) and composed of all 12 verified and complete chloroplast genomes that belong to the Boraginaceae family together with the plastome of *G. prostrata* subsp. *lusitanica* from this study. Concatenated nucleotide sequences coding for the shared proteome (75 coding sequences) extracted from the dataset were used in the phylogenetic analysis (see supplemental material for additional details). Four additional sequences were used as outgroups in the phylogenetic analysis: *Ehretia dicksonii* Hance (MZ555766.1; Xu et al. 2022) and *Tiquilia plicata* (Torr.) A.T.Richardson (MG573056.1; Schneider et al. 2018), belonging to the Boraginales order but not from the Boraginaceae family; *Salvia rosmarinus* Spenn. (NC_027259; Lamiaceae) also belonging to the lamiids clade; and *Tilia platyphyllos* Scop. (NC_062378; Malvaceae) from the malvids clade.

The IQ-TREE 2 software package (Minh et al. 2020) was used to analyze the MAFFT-aligned sequences (version 7.450, (Katoh and Standley 2013)) from the selected dataset. ModelFinder (Kalyaanamoorthy et al. 2017) determined TVM + F+I + I+R2 as the best-fit substitution model according to the Bayesian information criterion and, by using ultrafast bootstrap with UFBoot (10,000 replicates) (Hoang et al. 2018), IQ-TREE (Nguyen et al. 2015) reconstructed the corresponding tree.

## Results

**Figure 2.**
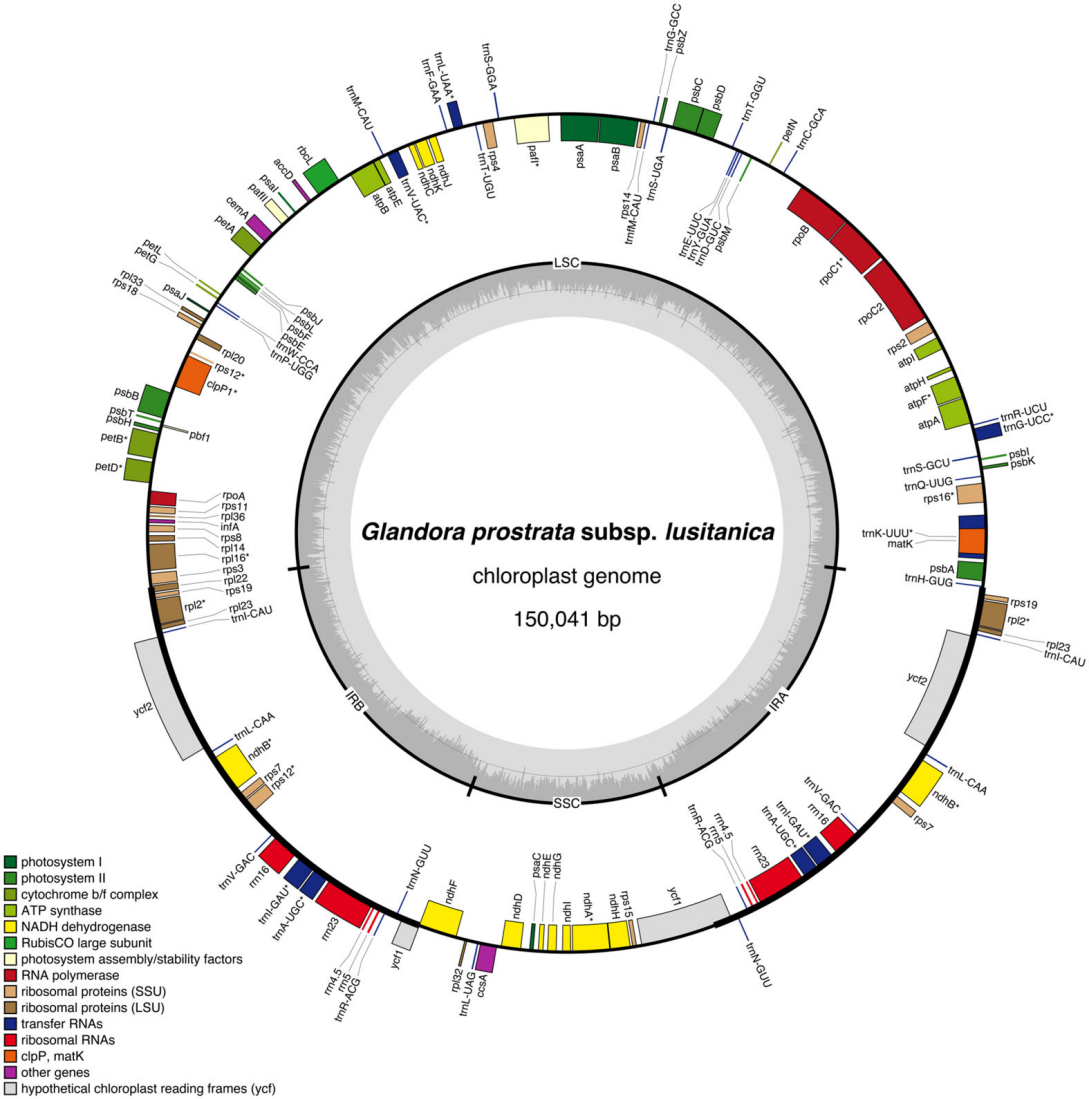
Graphical map of the complete chloroplast of *Glandora prostrata* subsp. *lusitanica* isolate BPTPS049 based on the conversion of annotations openly available in GenBank (accession number: ON641304), color coded based on their functional group, using OrganellarGenomeDRAW (OGDRAW) version 1.3.1 (Greiner et al. 2019). Genes inside the circle are transcribed clockwise, genes outside the circle counterclockwise, and intron-containing genes are marked by an asterisk (*). LSC: large single-copy region; SSC: small single-copy region; IRA, IRB: inverted repeats. The dark grey inner ring represents the GC content, while the complementary light grey ring represents the AT content.

The chloroplast genome of *G. prostrata* subsp. *lusitanica* isolate BPTPS049 (GenBank accession number: ON641304; Figure 2) is 150,041 bp in length with 37.5% GC content, displaying a quadripartite structure that contains a pair of inverted repeat (IR) regions (25,833 bp, GC content 43.0%), separated by a large single-copy (LSC) region (81,222 bp, GC content 35.4%) and a small single-copy (SSC) region (17,153 bp, GC content 31.0%). A total of 131 genes were predicted (114 of them unique), including 37 tRNA genes (30 of them unique), eight rRNA genes (four of them unique), and 86 protein-coding genes (80 of them unique).

**Figure 3.**
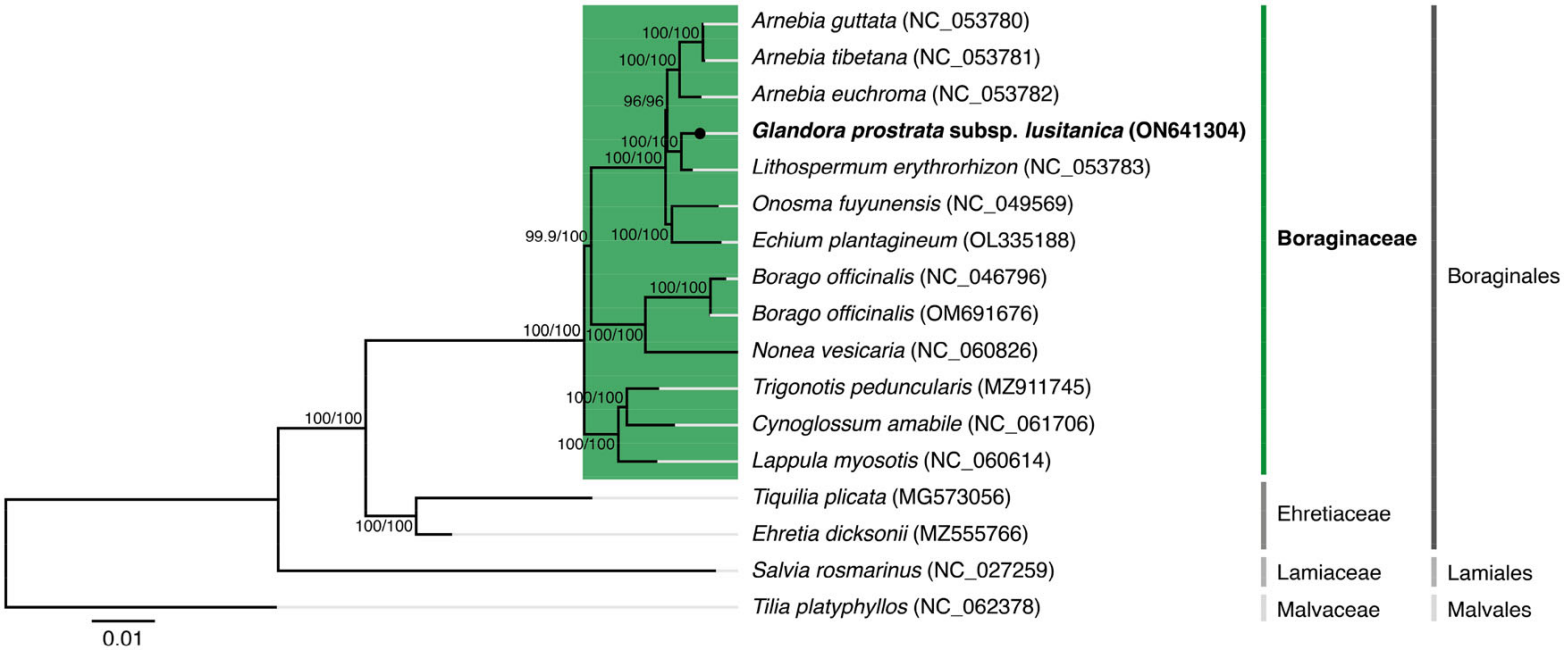
Maximum-likelihood tree inferred from the nucleotide sequences coding for the shared proteome from *Glandora prostrata* subsp. *lusitanica* isolate BPTPS049 and all 12 verified and complete chloroplast genomes belonging to the Boraginaceae family available in GenBank (accession date: 1 June 2022; see supplemental material for additional details). Numbers attached to the branches show the SH-aLRT and the UFBoot2 per cent supports (SH-aLRT/UFBoot2). *Ehretia dicksonii* (Ehretiaceae), *Tiquilia plicata* (Ehretiaceae), *Salvia rosmarinus* (Lamiaceae), and *Tilia platyphyllos* (Malvaceae) were used as outgroups to the Boraginaceae family (see supplemental material for additional details).

The maximum-likelihood tree obtained from the phylogenetic analysis performed (Figure 3) showed that *G. prostrata* subsp. *lusitanica* is situated under the Boraginaceae family, which belongs to the Boraginales order. A closer relationship with *Lithospermum erythrorhizon* Siebold & Zucc. is observable on the resulting tree with 100/100 per cent support (SH-aLRT/UFBoot2). When performing the phylogenetic analysis using the concatenated amino acid sequences of the shared proteomes (see supplemental material for additional details), the same tree results are supported.

## Discussion and conclusions

This study describes the chloroplast genome of *G. prostrata* subsp. *lusitanica* isolate BPTPS049, the first described plastome belonging to the *Glandora* genus. This complete genome will contribute to conservation, phylogenetic, and evolutionary studies that comprise this traditional species relevant to the landscape of aromatic, medicinal, and condiment plants from Portugal.

## Supplementary Material

Supplemental Material

Supplemental Material

## Data Availability

The data supporting this study are openly available in GenBank of NCBI at https://www.ncbi.nlm.nih.gov under the accession number ON641304. The NCBI BioProject and BioSample are PRJNA848680 and SAMN28118496, respectively. The ENA BioProject and SRA for the generated reads are PRJEB55314 and ERR10047929, respectively.
