# Supplementary material for: The complete plastome of Glandora prostrata subsp. lusitanica (Samp.) D.C.Thomas (Boraginaceae), the first chloroplast genome belonging to the Glandora genus
Source: Mitochondrial DNA B Resour. 2023 Feb 15;8(2):270–3. doi: 10.1080/23802359.2023.2175976 (PMC9937008; doi:10.1080/23802359.2023.2175976)
Supplement: Supplemental Material [file TMDN_A_2175976_SM9093.docx]

**Supplemental material to support the following publication:**

**The complete plastome of *Glandora prostrata* subsp. *lusitanica* (Samp.) D.C.Thomas (Boraginaceae), the first chloroplast genome belonging to the *Glandora* genus**

Inês CARVALHO LEONARDO^1,2^, Adriana ALBERTI^3,§^, France DENOEUD^3^, Maria Teresa BARRETO CRESPO^1,2^, Jorge CAPELO^4,5^, and Frédéric BUSTOS GASPAR^1,2,*^

^1^ iBET, Instituto de Biologia Experimental e Tecnológica, Apartado 12, 2781-901 Oeiras, Portugal

^2^ ITQB-NOVA, Instituto de Tecnologia Química e Biológica António Xavier, Universidade Nova de Lisboa, Av. da República, 2780-157 Oeiras, Portugal

^3^ Génomique Métabolique, Genoscope, Institut François Jacob, CEA, CNRS, Univ Évry, Université Paris-Saclay, 91057 Évry, France

^4^ ECOCHANGE, CIBIO-InBIO - Research Centre in Biodiversity and Genetic Resources, Universidade do Porto, Campus de Vairão, 4485-661 Vairão, Portugal

^5^ INIAV, Instituto Nacional de Investigação Agrária e Veterinária I.P., Quinta do Marquês, 2780-159 Oeiras, Portugal

^§^ Present address: Université Paris-Saclay, CEA, CNRS, Institute for Integrative Biology of the Cell (I2BC), 91198, Gif-sur-Yvette, France

* Corresponding author: fgaspar@ibet.pt

**PLANT MATERIAL ANALYSED**

**Figure S1**

Picture of voucher LISE: 96377 of *Glandora prostrata* subsp. *lusitanica* isolate BPTPS049 conserved at the LISE Herbarium (INIAV, Oeiras, Portugal; Jorge Capelo: [jorge.capelo@iniav.pt](mailto:jorge.capelo@iniav.pt); Identified by: Jorge Capelo). The plant material analysed (BioSample: SAMN28118496) in this study was collected from a wild population in the dunes of the Beja municipality (Vila Nova de Mil Fontes) in Portugal (Collection date: 2019-03-19; Location: 37.715 N 8.78361 W).


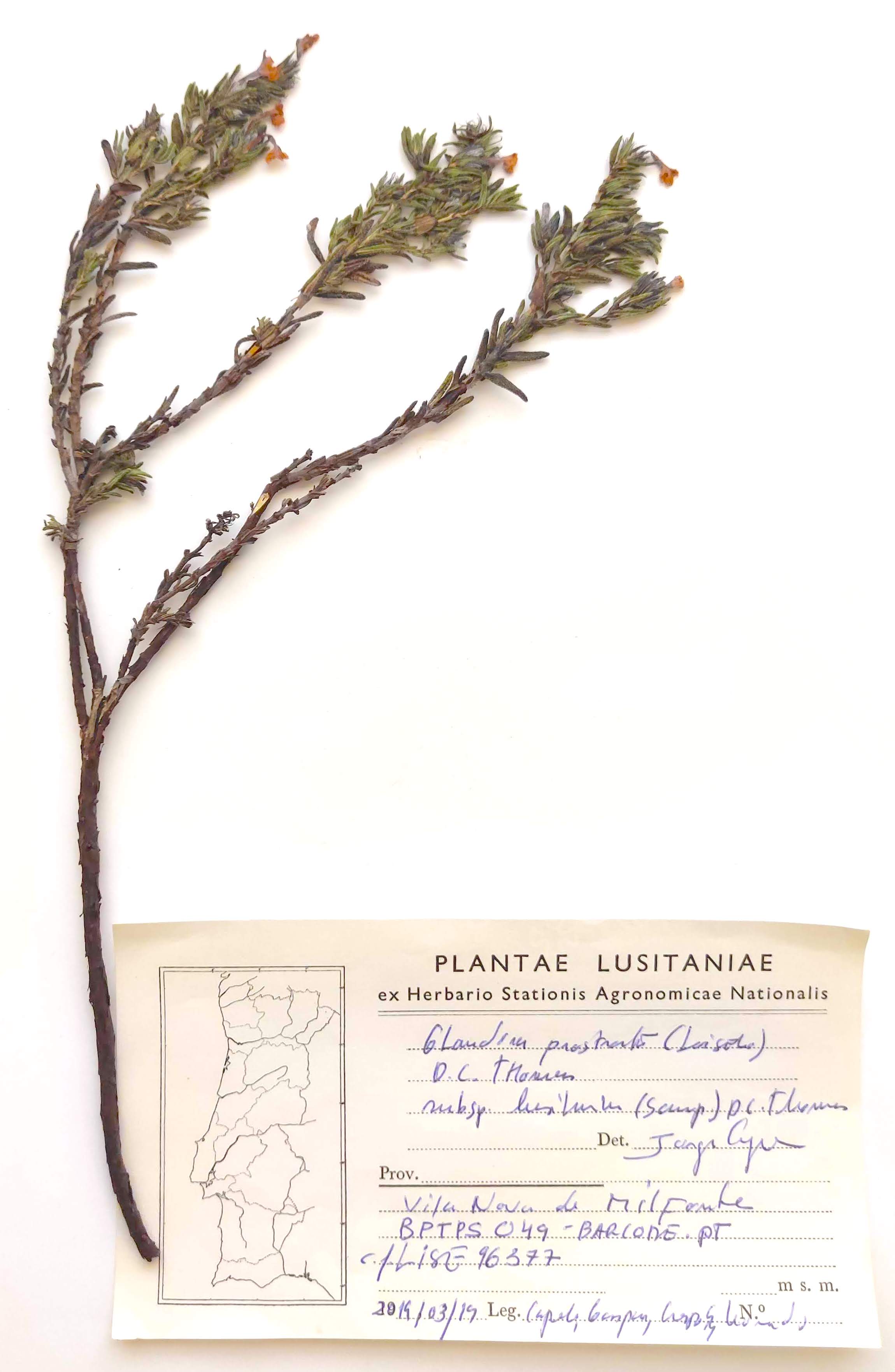


**PLASTOME ASSEMBLY AND EVALUATION**

**Assembly of the complete plastome of** ***Glandora prostrata* subsp. *lusitanica* isolate BPTPS049 using the GetOrganelle pipeline (v1.7.3.1)**

[ERR10047929](https://www.ncbi.nlm.nih.gov/sra/ERR10047929) is the SRA link where the reads used to assemble the complete plastome of *Glandora prostrata* subsp. *lusitanica* can be found. Forward and reverse reads are named CUE_AAADOSDA_4_1_HKFTKDSXY.12BA133_clean.fastq.gz and CUE_AAADOSDA_4_2_HKFTKDSXY.12BA133_clean.fastq.gz, respectively. The typical recipe suggested for Embryophyta plant plastome assembly (<https://github.com/Kinggerm/GetOrganelle>) using the GetOrganelle pipeline (v1.7.3.1) (Jin et al., 2020) was applied as described in the commands below while setting the flags “--max-reads” and “--reduce-reads-for-coverage” to 25 million and one thousand, respectively:

get_organelle_from_reads.py -1 CUE_AAADOSDA_4_1_HKFTKDSXY.12BA133_clean.fastq.gz -2 CUE_AAADOSDA_4_2_HKFTKDSXY.12BA133_clean.fastq.gz -o BPTPS049 -R 15 -k 21,45,65,85,105 -F embplant_pt --max-reads 25000000 --reduce-reads-for-coverage 1000

(Options description: “-1” - Input file with forward paired-end reads; “-2” - Input file with reverse paired-end reads; “-o” - Output directory; “-R” - Maximum number of extending rounds; “-F” - Target organelle genome type; “--max-reads” - Maximum number of reads to be used per file; “--reduce-reads-for-coverage” - Softbound for the maximum number of reads to be used)

**Assembly evaluation of the complete plastome of** ***Glandora prostrata* subsp. *lusitanica* isolate BPTPS049 using the mapping of reads**

As described by Jin et al. (2020), the script “evaluate_assembly_using_mapping.py” was used to evaluate the circular assembly obtained (see Figure S2 below).

It uses Bowtie2 to map reads to circular/non-circular assemblies; parses the SAM file; counts the number of mapped paired and unpaired reads; counts matched bases for each site (M), mismatched bases for each site (X), insertions between any two sites (I), and deletions for each site (D); and calculates the average and standard deviation of matched depth (ΣM and var(M)), average and standard deviation of mismatched depth (ΣX and var(X)), average and standard deviation of insertions between any two reference sites (ΣI and var(I)), and average and standard deviation of deletions per reference site (ΣD and var(D)) for each contig and the whole assembly. If ΣM > 0, a customized error rate would be also calculated as (ΣX+ΣI+ΣD)/ΣM with a customized deviation as (var(X) + var(I) + var(D))/ΣM.

**Table S1**

Table with the assembly quality and reads mapping statistics.

| Description | Counts |
| --- | --- |
| Mapped paired and unpaired reads | 1 381 143 × 2 + 19 589 |
| Mapped paired reads | 1 381 143 |
| Matched bases for each site (M)  (Average and standard deviation of matched depth (ΣM and var(M)) | 2 733.36 ± 287.24 |
| Mismatched bases for each site (X)  (Average and standard deviation of mismatched depth (ΣX and var(X)) | 6.62 ± 9.48 |
| Insertions between any two sites (I)  (Average and standard deviation of insertions between any two reference sites (ΣI and var(I)) | 0.41 ± 5.26 |
| Deletions for each site (D)  (Average and standard deviation of deletions per reference site (ΣD and var(D)) | 0.20 ± 2.88 |
| Customized error rate  ((ΣX+ΣI+ΣD)/ΣM with a customized deviation as (var(X) + var(I) + var(D))/ΣM) | 0.0026 ± 0.0042 |

**Figure S2**

Assembly quality and reads mapping plot using the “evaluate_assembly_using_mapping.py” script from the GetOrganelle pipeline (Jin et al., 2020). Each base of the assembly is on the X-axis; Reads counts for each base on the Y-axis.


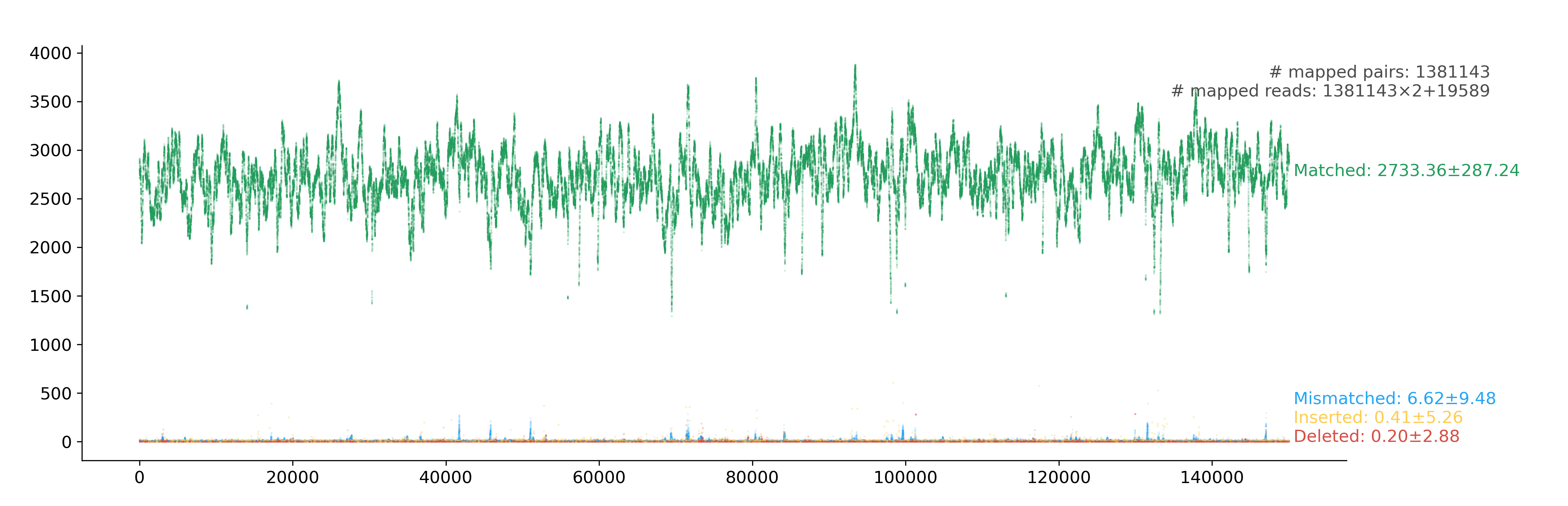


**PLASTOME ANNOTATION**

The GeSeq tool (Tillich et al., 2017) parameters used for the draft annotation of the complete plastome of *Glandora prostrata* subsp. *lusitanica* isolate BPTPS049 are described in Figure S3.

**Figure S3**

Screenshot of the GeSeq parameters used for the draft annotation of the complete plastome of *Glandora prostrata* subsp. *lusitanica* isolate BPTPS049


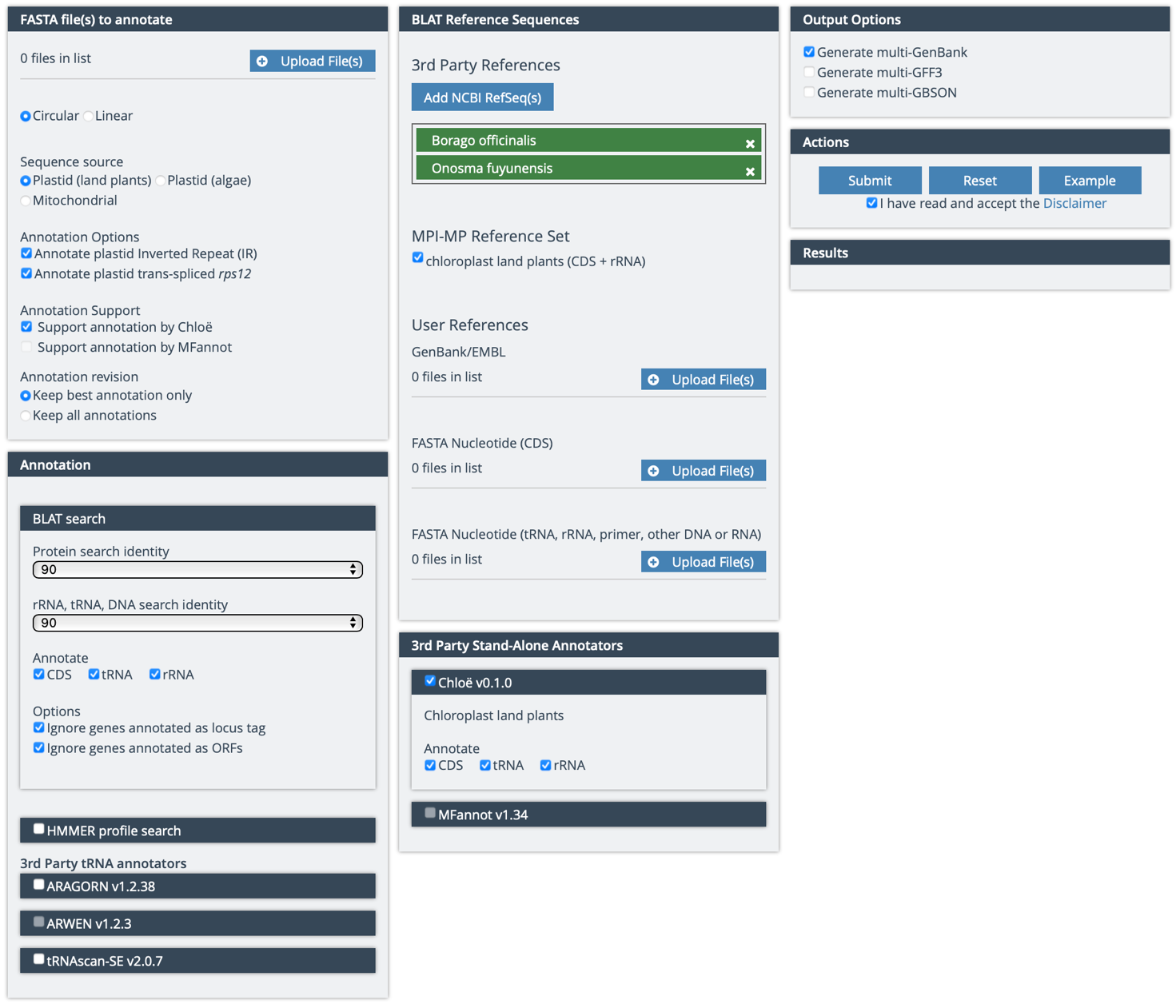


**PHYLOGENETIC ANALYSIS**

The phylogenetic analysis was performed using the concatenated nucleotide sequences coding for the shared proteome (75 coding sequences) extracted from a selected dataset. This dataset was composed of all 12 verified and complete chloroplast genomes belonging to the Boraginaceae family available in GenBank (Accession date: 2022-06-01) and the complete chloroplast genome of *G. prostrata* subsp. *lusitanica* obtained in this study. Four additional sequences were used as outgroups in the phylogenetic analysis: *Ehretia dicksonii* Hance (MZ555766.1) and *Tiquilia plicata* (Torr.) A.T.Richardson (MG573056.1), belonging to the Boraginales order but not from the Boraginaceae family; *Salvia rosmarinus* Spenn. (NC_027259; Lamiaceae) also belonging to the lamiids clade; and *Tilia platyphyllos* Scop. (NC_062378; Malvaceae) from the malvids clade.

The following list describes the complete chloroplast genomes used in this phylogenetic analysis (17 verified and complete chloroplast genomes, which comprises the outgroups), including the binomial nomenclature, GenBank accession number, formal publication reference, and the corresponding number of CDS described for each record.

The dataset used for the phylogenetic analysis presented in the manuscript was composed of all 12 verified and complete chloroplast genomes belonging to the Boraginaceae family available in GenBank (Accession date: 2022-06-01) and the complete chloroplast genome of *G. prostrata* subsp. *lusitanica* obtained in this study:

- *Arnebia euchroma* (NC_053782) (Park et al., 2020)
  - 88 CDSs
- *Arnebia guttata* (NC_053780) (Park et al., 2020)
  - 88 CDSs
- *Arnebia tibetana* (NC_053781) (Park et al., 2020)
  - 88 CDSs
- *Borago officinalis* (NC_046796) (no formal publication)
  - 89 CDSs
- *Borago officinalis* (OM691676) (no formal publication)
  - 87 CDSs
- *Cynoglossum amabile* (NC_061706) (no formal publication)
  - 84 CDSs
- *Echium plantagineum* (OL335188) (no formal publication)
  - 89 CDSs
- *Glandora prostrata* subsp. *lusitanica* (ON641304) (This study)
  - 86 CDSs
- *Lappula myosotis* (NC_060614) (no formal publication)
  - 83 CDSs
- *Lithospermum erythrorhizon* (NC_053783) (Park et al., 2020)
  - 88 CDSs
- *Nonea vesicaria* (NC_060826) (no formal publication)
  - 89 CDSs
- *Onosma fuyunensis* (NC_049569) (He et al., 2021)
  - 87 CDSs
- *Trigonotis peduncularis* (MZ911745) (Wu et al., 2022)
  - 86 CDSs

Four additional sequences were used as outgroups in the phylogenetic analysis:

- *Ehretia dicksonii* (MZ555766) (Xu et al., 2022)
  - 87 CDSs
- *Tiquilia plicata* (MG573056) (Schneider et al., 2018)
  - 86 CDSs
- *Salvia rosmarinus* (NC_027259) (no formal publication)
  - 87 CDSs
- *Tilia platyphyllos* (NC_062378) (no formal publication)
  - 84 CDSs

The phylogenetic analysis was performed using the following 75 concatenated nucleotide sequences, where no repeated genes were used, coding for the shared proteome extracted from the dataset described above:

*atpA, atpB, atpE, atpF, atpH, atpI, ccsA, cemA, clpP, infA, matK, ndhA, ndhB, ndhC, ndhD, ndhE, ndhF, ndhG, ndhH, ndhI, ndhJ, ndhK, pafI, pafII, petA, petB, petD, petG, petL, petN, psaA, psaB, psaC, psaI, psaJ, psbA, psbB, psbC, psbD, psbE, psbF, psbH, psbI, psbJ, psbK, psbL, psbM, psbN, psbT, psbZ, rbcL, rpl2, rpl14, rpl16, rpl20, rpl22, rpl32, rpl33, rpl36, rpoA, rpoB, rpoC1, rpoC2, rps2, rps3, rps4, rps7, rps11, rps12, rps14, rps15, rps16, rps18, rps19, ycf1*

The concatenated nucleotide sequences described above were aligned locally using the command line and MAFFT v7.505 (Katoh & Standley, 2013) using the automatic algorithm (with a 1.53 gap opening penalty).

mafft --auto glandora_prostrata_subsp_lusitanica_shared_CDS_nucleotide.fasta > glandora_prostrata_subsp_lusitanica_shared_CDS_nucleotide_mafft_aligned.fasta

The MAFFT alignment-containing file was further analysed with the IQ-TREE 2 software package (Minh et al., 2020) using the command line. The ultrafast bootstrap with UFBoot (Hoang et al., 2018) within the IQ-TREE 2 software package was performed using 10,000 replicates by running the following command:

iqtree2 -s glandora_prostrata_subsp_lusitanica_shared_CDS_nucleotide_mafft_aligned.fasta -alrt 10000 -B 10000

(Options description: “-s” - Input alignment file; “-alrt” - Replicates for SH approximate likelihood ratio test; “-B” - Replicates for ultrafast bootstrap)

The best-fit substitution model (TVM+F+I+I+R2 chosen according to the Bayesian Information Criterion) was selected according to ModelFinder (Kalyaanamoorthy et al., 2017), followed by a tree reconstruction (that is available in Figure 3 of the manuscript) using IQ-TREE (Nguyen et al., 2015).

The phylogenetic analysis was simultaneously performed using the concatenated amino acid sequences translated from the 75 nucleotide sequences coding for the shared proteome described above. Similarly, the concatenated amino acid sequences were aligned locally and further analysed with the IQ-TREE 2 software package (Minh et al., 2020) using the command line (ultrafast bootstrap performed using 10,000 replicates). The best-fit substitution model (JTT+F+I+I+R2 chosen according to the Bayesian Information Criterion) was selected according to ModelFinder (Kalyaanamoorthy et al., 2017), followed by a tree reconstruction (Figure S4) using IQ-TREE (Nguyen et al., 2015).

**Figure S4**

Maximum-likelihood tree inferred from the amino acid sequences of the shared proteome of *Glandora prostrata* subsp. *lusitanica* isolate BPTPS049 and all 12 verified and complete chloroplast genomes belonging to the Boraginaceae family available in GenBank (Accession date: 2022-06-01). Numbers attached to the branches show the SH-aLRT and the UFBoot2 per cent supports (SH-aLRT/UFBoot2). *Ehretia dicksonii* (Ehretiaceae), *Tiquilia plicata* (Ehretiaceae), *Salvia rosmarinus* (Lamiaceae), and *Tilia platyphyllos* (Malvaceae) were used as outgroups to the Boraginaceae family.


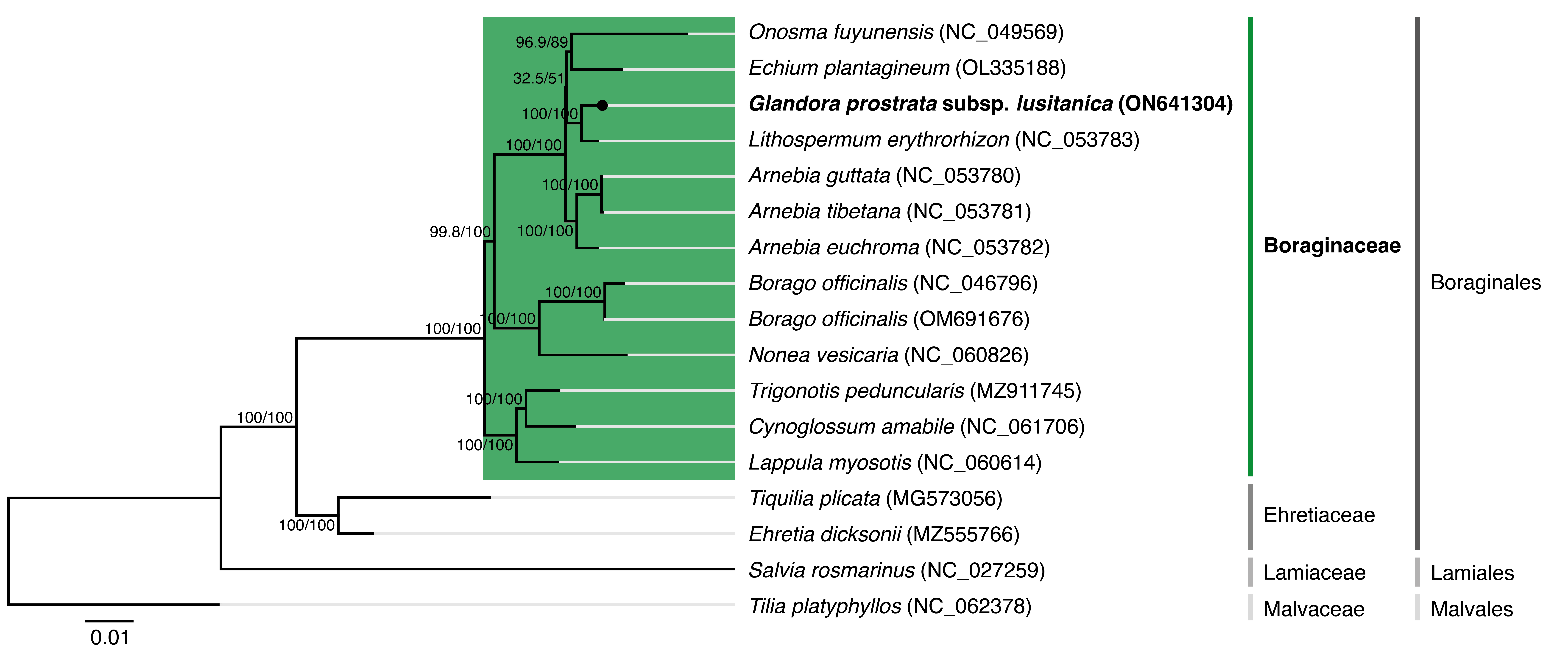


**REFERENCES**

He, Y., Xu, X., & Liu, Q. (2021). The complete chloroplast genome of *Onosma fuyunensis* Y. He & Q.R. Liu and its phylogenetic analysis. *Mitochondrial DNA. Part B, Resources*, *6*(11), 3142–3143. https://doi.org/10.1080/23802359.2020.1861567

Jin, J.-J., Yu, W.-B., Yang, J.-B., Song, Y., dePamphilis, C. W., Yi, T.-S., & Li, D.-Z. (2020). GetOrganelle: A fast and versatile toolkit for accurate de novo assembly of organelle genomes. *Genome Biology*, *21*(1), 241. https://doi.org/10.1186/s13059-020-02154-5

Park, I., Yang, S., Song, J.-H., & Moon, B. C. (2020). Dissection for Floral Micromorphology and Plastid Genome of Valuable Medicinal Borages *Arnebia* and *Lithospermum* (Boraginaceae). *Frontiers in Plant Science*, *11*, 606463. https://doi.org/10.3389/fpls.2020.606463

Schneider, A. C., Braukmann, T., Banerjee, A., & Stefanović, S. (2018). Convergent Plastome Evolution and Gene Loss in Holoparasitic Lennoaceae. *Genome Biology and Evolution*, *10*(10), 2663–2670. https://doi.org/10.1093/gbe/evy190

Tillich, M., Lehwark, P., Pellizzer, T., Ulbricht-Jones, E. S., Fischer, A., Bock, R., & Greiner, S. (2017). GeSeq – versatile and accurate annotation of organelle genomes. Nucleic Acids Research, 45(W1), W6–W11. https://doi.org/10.1093/nar/gkx391

Wu, J.-H., Li, H.-M., Lei, J.-M., & Liang, Z.-R. (2022). The complete chloroplast genome sequence of *Trigonotis peduncularis* (Boraginaceae). *Mitochondrial DNA. Part B, Resources*, *7*(3), 456–457. https://doi.org/10.1080/23802359.2022.2048212

Xu, X., Cheng, Y., Tong, L., Tian, L., & Xia, C. (2022). The complete chloroplast genome sequence of *Ehretia dicksonii* Hance (Ehretiaceae). *Mitochondrial DNA. Part B, Resources*, *7*(4), 661–662. https://doi.org/10.1080/23802359.2022.2061873
